# Adaptive radiotherapy dose prediction on head and neck cancer patients with a 3D multi-headed U-Net deep learning architecture

**DOI:** 10.1088/3049-477X/adfade

**Published:** 2025-08-26

**Authors:** Hui-Ju Wang, Austen Maniscalco, David Sher, Mu-Han Lin, Steve Jiang, Dan Nguyen

**Affiliations:** Medical Artificial Intelligence and Automation Laboratory, Department of Radiation Oncology, University of Texas Southwestern Medical Center, Dallas, TX, United States of America

**Keywords:** artificial intelligence, dose prediction, adaptive radiation therapy, head and neck cancer

## Abstract

Online adaptive radiation therapy (ART) personalizes treatment plans by accounting for daily anatomical changes, requiring workflows distinct from conventional radiotherapy. Deep learning-based dose prediction models can enhance treatment planning efficiency by rapidly generating accuracy dose distributions, reducing manual trial-and-error and accelerating the overall workflow; however, most existing approaches overlook critical pre-treatment plan information—specifically, physician-defined clinical objectives tailored to individual patients. To address this limitation, we introduce the multi-headed U-Net (MHU-Net), a novel architecture that explicitly incorporates physician intent from pre-treatment plans to improve dose prediction accuracy in adaptive head and neck cancer treatments. Our dataset comprised 43 patients, each with pre-treatment plans, adaptive treatment plans, structure sets, and CT images. MHU-Net builds upon the widely adopted Stander U-Net architecture, extending it with a dual-head design: the primary head processes adaptive session contours and their corresponding signed distance maps, while the secondary head integrates pre-treatment contours, signed distance maps, and dose distributions. The features are merged within a primary U-Net framework to enhance dose prediction accuracy for adaptive treatment sessions. To evaluate the effectiveness of MHU-Net, we conducted a comparative analysis against U-Net. On average, MHU-Net reduced organ-at-risk dose prediction errors, achieving 1.78% lower maximum dose error and 1.22% lower mean dose error compared to U-Net. For the planning target volume, MHU-Net demonstrated significantly improved accuracy, with maximum and mean dose errors of 3.54  ±  2.75% and 1.07 ± 0.88%, respectively, outperforming U-Net’s corresponding errors of 5.36 ± 4.19% and 2.76 ± 2.22% (*P* < 0.05). Taken together, these findings demonstrate that the proposed MHU-Met advances DL-based dose prediction for ART by effectively integrating both pre-treatment and adaptive session data. This approach facilitates the generation of dose distributions that more closely resemble the clinical ground truth, supporting personalization in ART planning and improving alignment with physician intent and treatment objectives.

## 1. Introduction

During external beam radiotherapy (RT), head-and-neck (H&N) cancer patients often experience weight loss and anatomical changes, such as the location, shape, and size of the tumor and surrounding organs. These changes introduce uncertainties, leading to larger treatment areas and increased side effects [1–3]. Adaptive radiotherapy (ART) has emerged as a cutting-edge solution to address these challenges by integrating advanced imaging, adaptation assessment, treatment planning, and quality assurance to monitor inter- and intra-fraction anatomical variations of the target and organs-at-risk (OARs) [4, 5].

A notable advancement is online ART, where patients remain on the couch from imaging to treatment delivery, enabling daily re-planning with MRI and cone beam computed tomography (CT) guidance to correct positioning errors, adapt to anatomical changes, improve target localization, minimize healthy tissue exposure, and enhance tumor control [5–9]. However, its planning workflow differs significantly from conventional methods. Its tasks often involve re-simulation, re-contouring, and re-planning while the patient holds their treatment position on the couch, which increases both treatment time and clinical workload [10, 11]. Although planning efficiency is improved through the use of automation tools like auto-contouring, knowledge-based treatment planning, and verification automation, delays continue because of contouring adjustments and the suboptimal quality of automated dose distribution calculations, necessitating more review time [12]. Furthermore, the planning process becomes more complex due to multiple dose levels and numerous OARs, as in the case of H&N RT.

To address this, the integration of artificial intelligence (AI), especially deep learning (DL) algorithms, has emerged as a significant development. Dose prediction models are one of the AI applications in RT that are used to maintain high-quality planning for patients and reduce planning time and resources. Convolutional neural networks (CNNs), a specialized branch of DL, have proven particularly effective in image-processing tasks. CNN-based dose prediction models excel at automatically extracting features and learning complex mapping relationships from patients’ anatomical data on CT images and region-of-interest masks to predict accurate dose distribution maps [13, 14]. Building on this foundation, more advanced CNN-based approaches have emerged to improve volumetric dose prediction by leveraging enhanced network architectures, including the hierarchically densely connected U-Net [15], dilated convolutional U-Net [16], and cascaded U-Net [17]. Despite ongoing research on the clinical applications of DL-based models, their integration into the emerging ART treatment modality has remained relatively unexplored, primarily due to its novel workflow and limited data availability.

Recognizing this gap in adaptation, more recent research has started looking at AI-driven alternatives to improve ART workflow [18–22]. A publication by Chun *et al* [19] in 2021 proposed integrating patient-specific data into DL model training for re-planning CT auto-contouring using the ‘intentional deep overfit learning’ (IDOL) approach, which fine-tunes a generalized model for individual patients. Inspired by this, Maniscalco *et al* [20] applied the IDOL method to refine a population-based DL model with patient-specific data, enhancing ART dose prediction, improving plan quality, reducing the treatment planning burden, and streamlining adaptive sessions compared to population models trained solely on adaptive data. However, fine-tuning a patient-specific model to generate a single dose prediction result requires approximately 1 h, whereas population models—widely used in both research and commercial settings—can be applied instantly once the pre-treatment plan is updated. Despite their efficiency, current adaptive population models rely only on adaptive session data, lacking prior knowledge of a patient’s initial dose distribution [19, 21, 22].

To address this limitation, we aim to develop the customized multi-headed input U-Net (MHU-Net) based population dose prediction model for H&N online ART, integrating comprehensive pre-treatment and adaptive plan data. Making precise adaptive session dose predictions is essential for maintaining treatment effectiveness and achieving optimal patient outcomes, especially when tumors and OARs frequently undergo significant changes in volume, shape, and position. By incorporating physician- and patient-specific intents, the goal of MHU-Net is to ensure that key clinical considerations are carried forward into subsequent adaptive sessions as anatomical changes occur without training patient-specific models, maintaining the rapid applicability of traditional adaptive population models while improving prediction accuracy and alignment with clinically approved ground truth plans. We hypothesize that MHU-Net will generate more accurate adaptive dose predictions by leveraging richer input information and incorporating multi-head input mechanisms, in contrast to the standard U-Net, which is trained solely on adaptive plans.

## 2. Materials and methods

### 2.1. H&N cancer patient dataset

This retrospective study included clinical data from 43 H&N cancer patients treated with Varian Ethos machines. This study protocol was approved by the institutional review board. Initially, treatment planning for these patients was based on a pre-treatment CT scan, which established the foundation for their radiotherapy strategy, incorporating key clinical goals and priorities. Throughout the course of treatment, daily ART plans were created using synthetic CT images. These were generated by deformably registering the initial planning CT to each day’s cone-beam CT, aligning it to daily anatomy for accurate adaptation to anatomical changes. These synthetic CTs were created automatically within the Varian Ethos™ treatment-planning environment (Varian Medical Systems, Palo Alto, CA) using its built-in deformable image registration algorithm. A physician reviewed the structures on each synthetic CT after registration.

All the patient data included dose distributions from the pre-treatment and adaptive plans, along with their associated images, OARs and planning target volumes (PTVs). The physician-approved dose distributions from adaptive sessions served as the ground truth in this study, providing a benchmark for evaluating the accuracy of our predictive models. Our patient cohort included individuals with 1–5 PTVs, who received prescription doses ranging from 42.5 to 72 Gy. A total of 44 OARs were identified, including the body, brachial plexus (left and right), brain, brainstem, cerebellum (left and right), cochlea (left and right), constrictor muscles (superior, middle, inferior, and combined), esophagus, larynx, mandible, masseter muscles (left, right, and combined), oral cavity, post-arytenoid & cricoid space, parotid glands (left, right, and combined), submandibular glands (left and right), lacrimal glands (left and right), optic nerves (left and right), optic chiasm, optic pathway, eyes (left and right), thyroid, spinal cord, spinal canal, soft palate, epiglottis, temporal lobes (left and right), and superior parotid glands (left and right).

### 2.2. Data preprocessing

The data had been preprocessed by extracting CT scans, structure contours, and approved dose DICOM files from Eclipse TPS and converting them to NumPy arrays. To reduce video random access memory consumption during model training, the voxel resolution of the contours and dose was standardized to 5 × 5 × 5 mm^3^. PTVs were merged into a single array in units of Gy with each voxel assigned a value based on the corresponding PTV. In cases where multiple PTVs overlapped within the same voxel, the highest prescription dose among them was used. Voxels without any PTV were set to zero. Each OAR was preprocessed into a binary mask, for which a voxel value of 0 indicated OAR absence and 1 indicated OAR presence. We also incorporated signed distance maps alongside binary masks as input, where each voxel encodes its Euclidean distance to the target’s isocenter, with values of 1.0 at the PTV and decreasing toward 0.0 as distance increases. Distance maps, supported by efficient algorithms in many image processing applications [23], have been used to enhance spatial reasoning in dose prediction [24–26] distance map was introduced as additional network input to handle differences in CT resolutions and tumor lengths, and to describe CT shifts. The final input to MHU-Net consists of 181 channels in total. The primary head receives 90 channels, comprising 44 adaptive OAR masks, 44 corresponding OAR distance maps, the PTV, and the deformed CT from the adaptive session. The secondary head takes 91 channels, including the original pre-treatment planning data: 44 OAR masks, 44 OAR distance maps, the PTV, the original CT, and the physician-approved dose distribution.

### 2.3. Deep learning model architecture

To effectively integrate pre-treatment and adaptive data for accurate adaptive session dose prediction, we designed MHU-Net, an advanced U-Net extension with a novel dual-head architecture. The primary input head processes adaptive session anatomy, while the secondary input head integrates initial CT and RT dose distributions.

**Figure 1.**
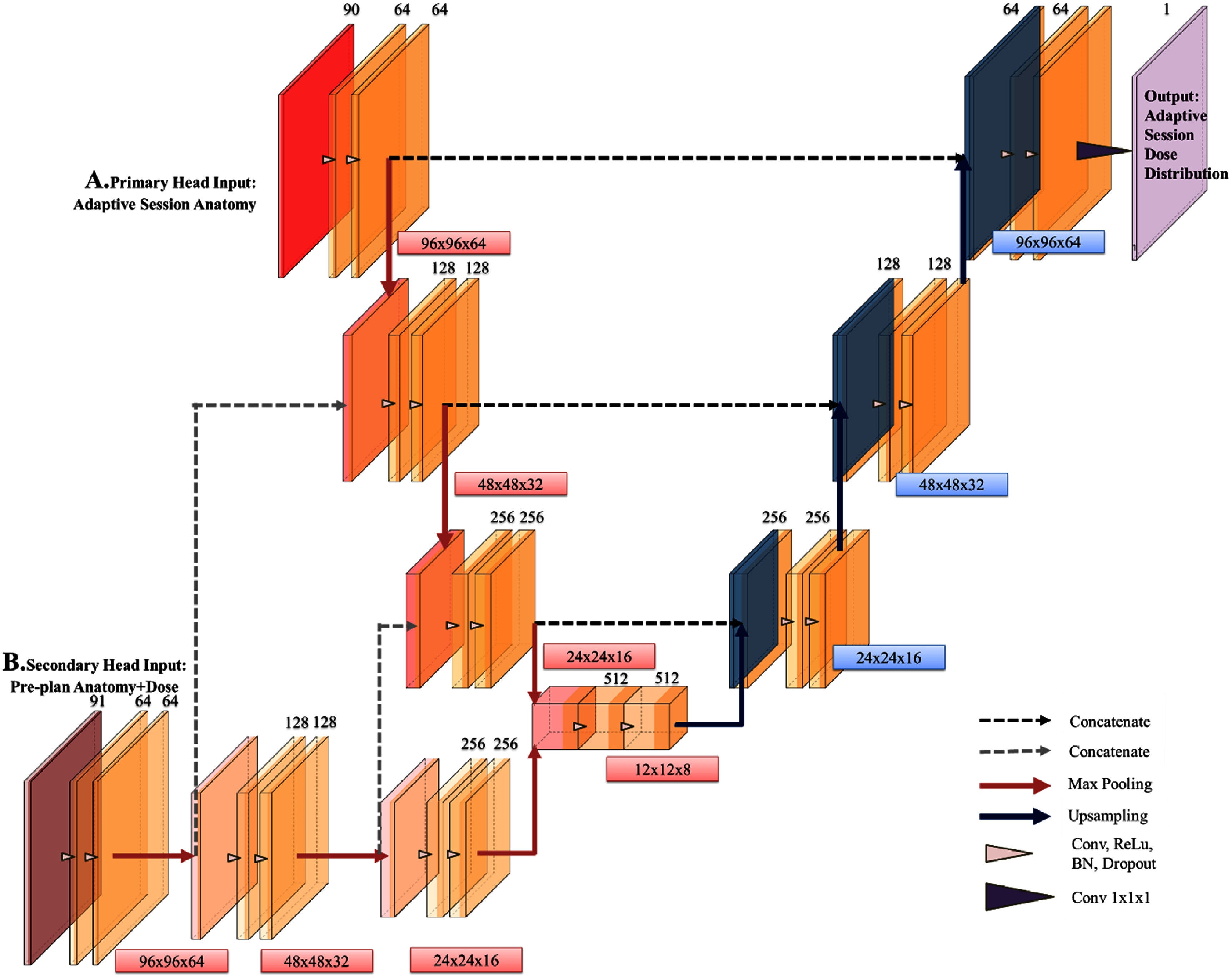
Schematic of the proposed MHU-Net architecture. The numbers above the boxes represent the number of features for each map, while the numbers to the right of each hierarchy in the MHU-Net represent the corresponding matrix dimension. *Diagram generated with PlotNeuralNet.*

As shown in figure 1, the primary head input (figure 1(A)) processes adaptive session contours and their signed distance maps. It begins with an encoder block, where each level includes two convolutional layers and a downsampling layer. Each layer employs 3D convolutions with 3 × 3 × 3 kernels, a stride of 1, and ReLU activation to capture complex, non-linear patterns in the dose distribution. Group normalization with 32 groups is used to accelerate convergence and improve generalization. To mitigate overfitting, we applied block dropout regularization using a of 0.05 and a dropout power of 0.25, based on the formulation: *dropout* rate = maximum dropout rate × ($\frac{{{\text{current}}\_{\text{num}}\_{\text{filters }}}}{{{\text{max}}\_{\text{num}}\_{\text{filters}}}}$)^dropout power^. This method provides stronger regularization in the deeper layers, where the number of filters is higher. After the convolutional layers, downsampling is performed through 3D max pooling and 3D average pooling with 2 × 2 × 2 kernels, effectively reducing the spatial dimensions by half while preserving essential features such as edges and textures. For example, the first layer has a feature map of shape [1, 64, 96, 96, 64] with a batch size of 1 and 64 initial input channels. The output after max pooling has a shape of [1, 128, 48, 48, 32]. This process of convolution, followed by downsampling, is repeated for multiple levels.

At the same time, the secondary input head is specifically designed to process pre-treatment contours, signed distance maps, and dose distribution data, ensuring alignment with the original treatment strategy. Similar to the primary-headed input, the secondary head (figure 1(B)) mirrors the primary head’s four-level downsampling structure with the same number of convolution and pooling layers. Feature maps from the same encoder layer in the secondary head are copied and concatenated with those in the primary head, enabling cross-stream information exchange. For example, the first encoding layer in both heads generates tensors with a shape of [1, 64, 96, 96, 64], producing 64 feature maps. These are then concatenated with the second layer of the primary head before undergoing additional 3D convolutional operations and max pooling in the second encoding layer. Next, the secondary head produces a tensor with a shape of [1, 128, 48, 48, 32] after 3D convolutional operations, which is again concatenated across both heads, continuing the progressive fusion of features from the pre-treatment plan and adaptive data. After the third max-pooling layer, both heads merge into a final encoding layer with two additional convolutions, forming a bottleneck tensor [1, 512, 12, 12, 8]. This bottleneck is the critical convergence point, integrating real-time anatomical changes from the adaptive session with pre-treatment planning constraints, ensuring robust and context-aware dose prediction.

Beyond the bottleneck, the up-sampling path (right side) consists of four decoding layers, following the same structure as U-Net. Each layer comprises an upsampling operation followed by two 3 × 3 × 3 convolutional layers and ReLU. The upsampling layer consists of two distinct operations that increase the spatial dimensions of the feature maps. The first upsampling operation employs nearest-neighbor interpolation. This method enlarges the spatial dimensions by assigning each pixel in the upsampled map the value of the nearest pixel in the original map, avoiding any blending of neighboring values. The second operation uses trilinear interpolation, calculating new pixel values by taking a weighted average of the surrounding voxels in 3D space. This method provides smoother transitions between pixel values. The outputs from both upsampling operations, along with those from the transposed convolutional layer, are concatenated and passed to the convolutional layers. At this stage, one feature map flows through the shared decoder with skip connections, merging its representations with those from the encoder at the same level before the convolution operations. The other feature map bypasses these connections, adjusting its size after upsampling to ensure consistent shapes. This process of upsampling and concatenation continues through the remaining levels until the final output layer. A 1 × 1 × 1 convolution refines the final output to [1, 1, 96, 96, 64], ensuring spatial alignment with input data.

**Figure 2.**
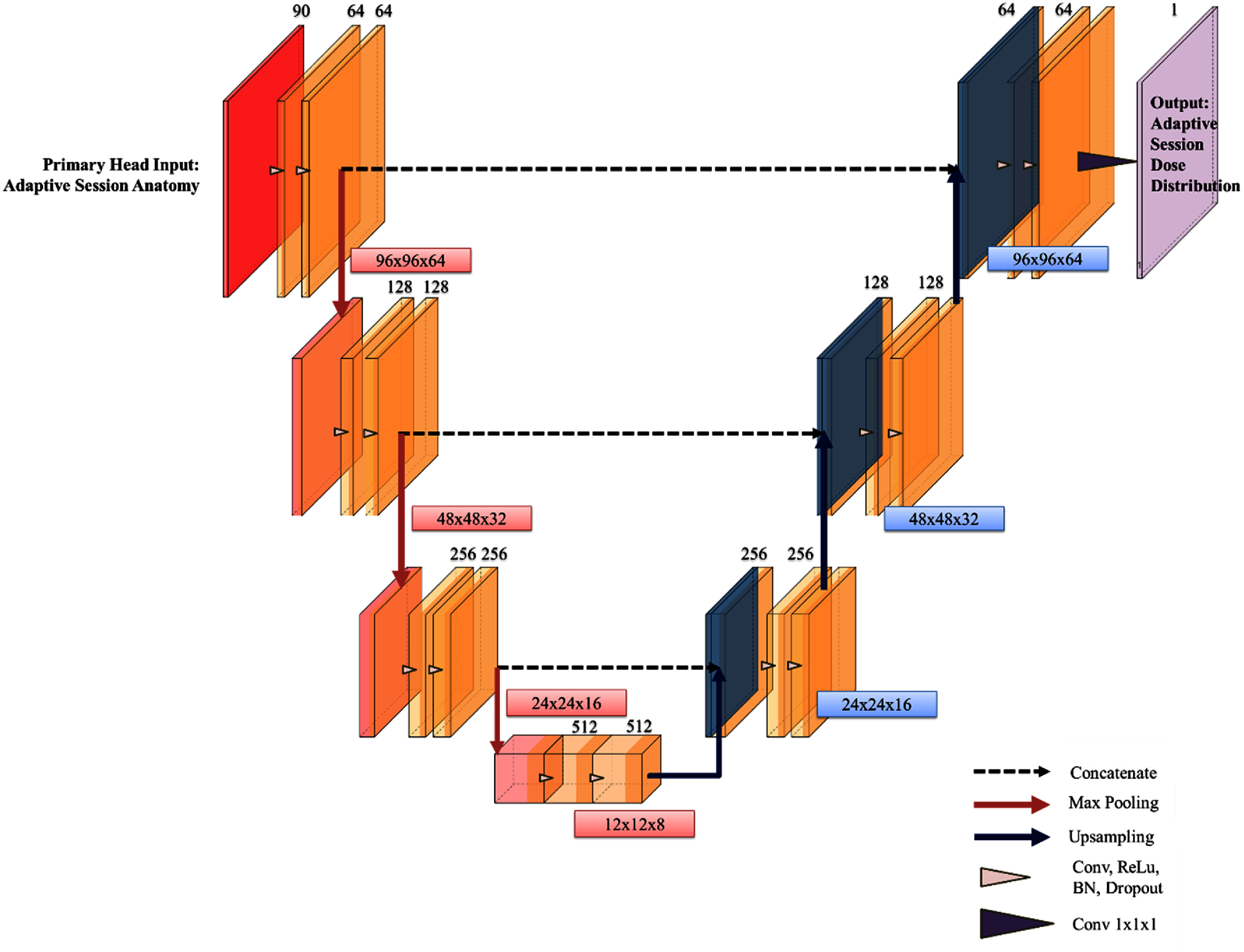
Schematic of the baseline U-Net architecture. The numbers above the boxes represent the number of features for each map, while the numbers to the right of each hierarchy in the U-Net represent the corresponding matrix dimension.

To evaluate our implementation, we compared MHU-Net with U-Net as shown in figure 2. The input of U-Net takes 44 adaptive OARs, 44 OAR distance maps, OAR distance maps, the PTV, and the deformed CT from the adaptive sessions. For a fair comparison, U-Net was constructed with the same number of downsampling operations as MHU-Net, following the conventional U-Net structure where the number of filters doubles after each max-pooling operation. The key difference is that U-Net lacks the secondary head input and processing path for pre-treatment plan data.

### 2.4. Training and validation

Since U-Net relied solely on adaptive session data to predict adaptive dose distributions, its training dataset consisted of individual patient ART data [5–8]. This included 55 dose distributions from adaptive session plans across 30 patients for training and 7 dose distributions from 3 patients for validation. In contrast, MHU-Net was trained on a broader dataset that included both pre-treatment and adaptive session dose distributions, totaling 85 dose distributions (30 from pre-treatment plans and 55 from adaptive session plans) across 30 patients. The validation set included 7 dose distributions from adaptive session plans across 3 patients.

To manage GPU memory limitation, we used random 96 × 96 × 64 patches as input for training, with a batch size of 1 sample for efficient processing. Data augmentation was implemented during the patch generation process through random translations, flips, and in-plane rotations, restricted to angles of 0°, 90°, 180°, or 270°. Translations were executed by sampling the patch center from a Gaussian distribution centered on the center of mass of the PTV. Flips and rotations were applied independently, each with a probability of 50%. We coded the DL based model using the TensorFlow package and trained models using an NVIDIA V100 GPU with 32 GB of memory. Model training used the Adam optimizer with a constant learning rate of 1 × 10^−4^. The loss function was mean squared error (MSE), calculated by summing the squared voxel-wise differences between the model’s predicted dose and actual dose distribution and then dividing by the total number of voxels. Models were trained over 3000 epochs, with the best-performing weights saved at the checkpoint that had the lowest validation loss, which was selected for evaluation.

### 2.5. Evaluation

To assess model performance, we first calculated the absolute percent error for the mean and maximum dose ($\frac{{{{\boldsymbol{D}}_{{\mathbf{Ground}} \,\,{\mathbf{Truth}}}} - {{\boldsymbol{D}}_{{\mathbf{Predicted}}}}}}{{{{\boldsymbol{D}}_{{\mathbf{Highest}}{ }\,\,{\mathbf{Presciption}}{ }}}}}{\text{}} *100$), where the highest prescription refers to the maximum prescribed dose within each individual patient’s treatment plan. The clinical treatment plan served as the reference. For individual PTVs, we focused on the highest dose PTV in each plan, evaluating metrics like *D*_99%_, *D*_98%_, *D*_95%_, and homogeneity ($\frac{{{{\mathbf{D}}_{2\% }} - {{\mathbf{D}}_{98\% }}}}{{{{\mathbf{D}}_{50\% }}}}$) to compare model predictions with the ground truth. With a significance level of 0.05, we used the two-tailed Wilcoxon signed-rank test to assess differences between MHU-Net and U-Net, as our dataset violates the normality assumption required for a paired *t*-test. Additionally, we examined spatial dose distributions and generated DVHs from the test patient pool, analyzing differences in DVH metrics to assess the agreement between predicted and ground truth doses.

## 3. Results

In a study with 10 test patients, although both models exhibited similar training loss trends during development, MHU-Net maintained a more stable and consistently lower validation loss. It achieved a MSE of 0.0038 ± 0.0013, which is lower than the 0.0048 ± 0.0014 observed by U-Net, with a statistically significant *p*-value of 0.006 in a paired *t*-test. This result underscores MHU-Net is better at generalizing unseen data from mapping accurate dose predictions to the contour.

**Figure 3.**
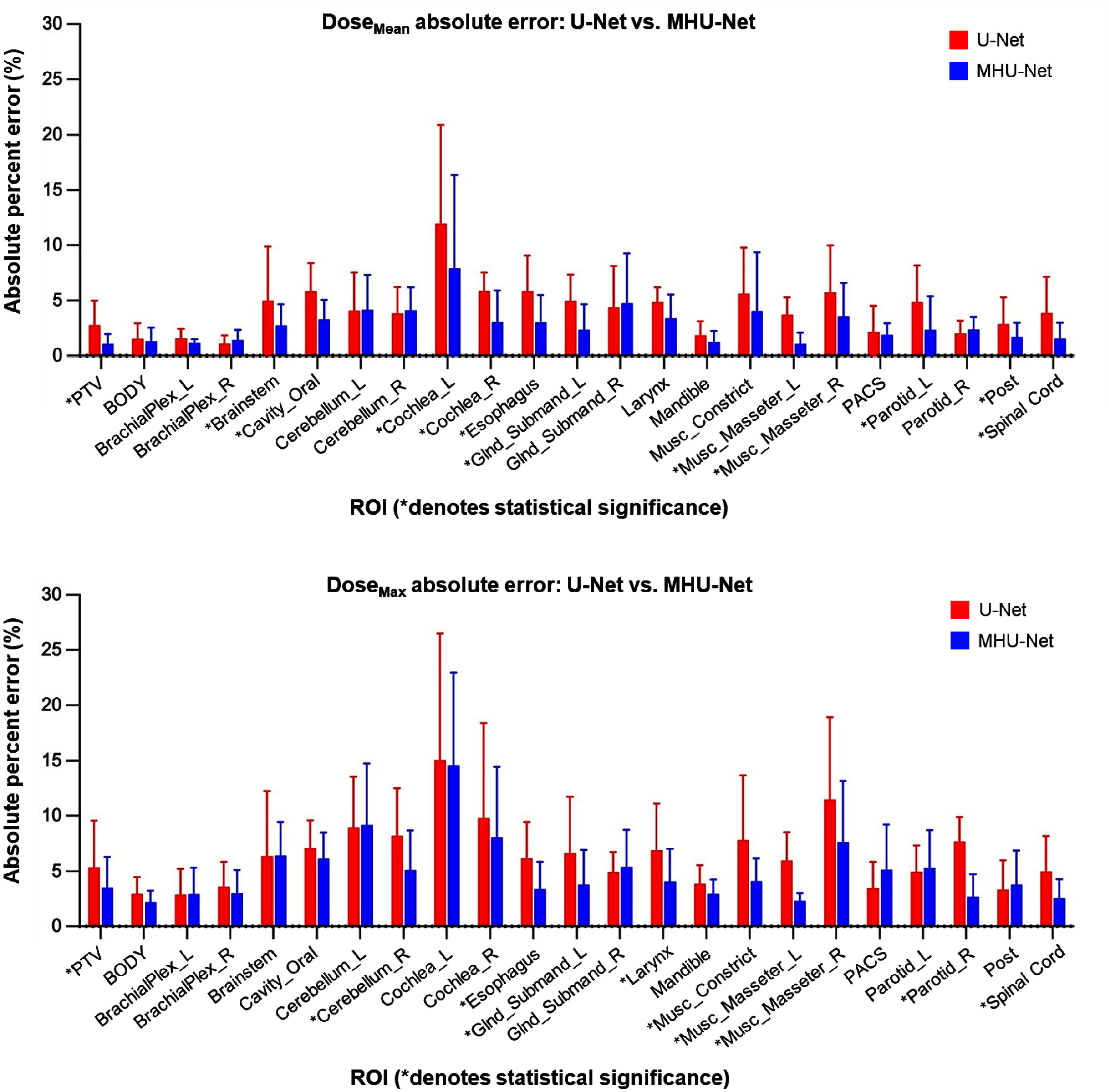
Absolute percent error of mean dose (*D*_mean)_ and maximum dose (*D*_max)_ for the structures of interest. Error is reported as a percentage of the prescription dose. Statistically significant differences are marked with an asterisk (*) for *p* < 0.05.

Figure 3 shows the comparison of dose prediction models based on the maximum and mean dose error (*D*_mean_ and *D*_max_) for each predicted structure, measured against the ground truth. The results are averaged across all 10 adaptive treatment plans. We evaluated differences in *D*_mean_ and *D*_max_ between the two models using a two-tailed Wilcoxon signed-rank test with an alpha of 0.05 across all 22 individual OARs. Figure 3 shows that in the *D*_mean_ absolute percent error analysis, 17 out of 22 structures exhibit lower *D*_mean_ errors with MHU-Net (red bar) compared to U-Net (blue bar). Several high-impact OARs, including brainstem, oral cavity, left cochlea, right cochlea, esophagus, left submandibular gland, left masseter muscle, right masseter muscle, left parotid gland, spinal cord, and posterior neck region, demonstrate significantly reduced *D*_mean_ errors with MHU-Net. Similarly, the absolute percent error for *D*_max_ reveals that 15 out of the 22 structures have lower dose prediction errors from MHU-Net, compared to predictions made by U-Net. Significant reductions in *D*_max_ prediction errors are observed for the right cerebellum, esophagus, left submandibular gland, larynx, constrictor muscle, left masseter muscle, right masseter muscle, right parotid gland, and spinal cord when using MHU-Net. Not all lower-dose prediction errors show statistically significant results, which may be due to the small sample size. When averaged across all OARs, MHU-Net predicted the OAR *D*_max_ prediction errors within 5.05% and the *D*_mean_ prediction errors within 3.22% of the prescription dose for the 10 test patients. For comparison, U-Net yielded corresponding errors of 6.83% and 4.44%. By reducing both *D*_mean_ and *D*_max_ prediction errors, MHU-Net has the ability to guides ART adaptive session planning to minimize radiation exposure to sensitive structures.

Figure 3 also shows that *D*_mean_ and *D*_max_ errors for the PTV dose prediction are significantly lower in MHU-Net as compared to U-Net. In our analysis of PTV dose prediction in table 1, we assessed the average percent prediction error for the highest dose PTV in each treatment plan, comparing it to the ground truth using metrics such as *D*_95%_, *D*_98%_, *D*_99%_, and homogeneity. We found that MHU-Net achieves statistically significantly lower error in predicting the dose coverage and homogeneity on the test set, as compared to U-Net.

**Table 1.** Comparative mean absolute error average (± SD) dosimetric results in the highest dose PTV in 10 tested patients.

PTV Dosimetric index	U-Net (%)	MHU-Net (%)	*p*-value
*D*_max_	5.36 ± 4.14	3.54 ± 2.75	0.019
*D*_mean_	2.76 ± 2.23	1.06 ± 0.88	0.031
*D*_99%_	3.65 ± 1.41	2.75 ± 1.39	0.019
*D*_98%_	5.18 ± 2.06	2.95 ± 1.50	0.007
*D*_95%_	5.94 ± 2.54	3.94 ± 2.43	0.027
Homogeneity	5.34 ± 2.34	2.57 ± 1.46	0.002

*Note:* D_n%_ means the dose received by n % of the PTV.

**Figure 4.**
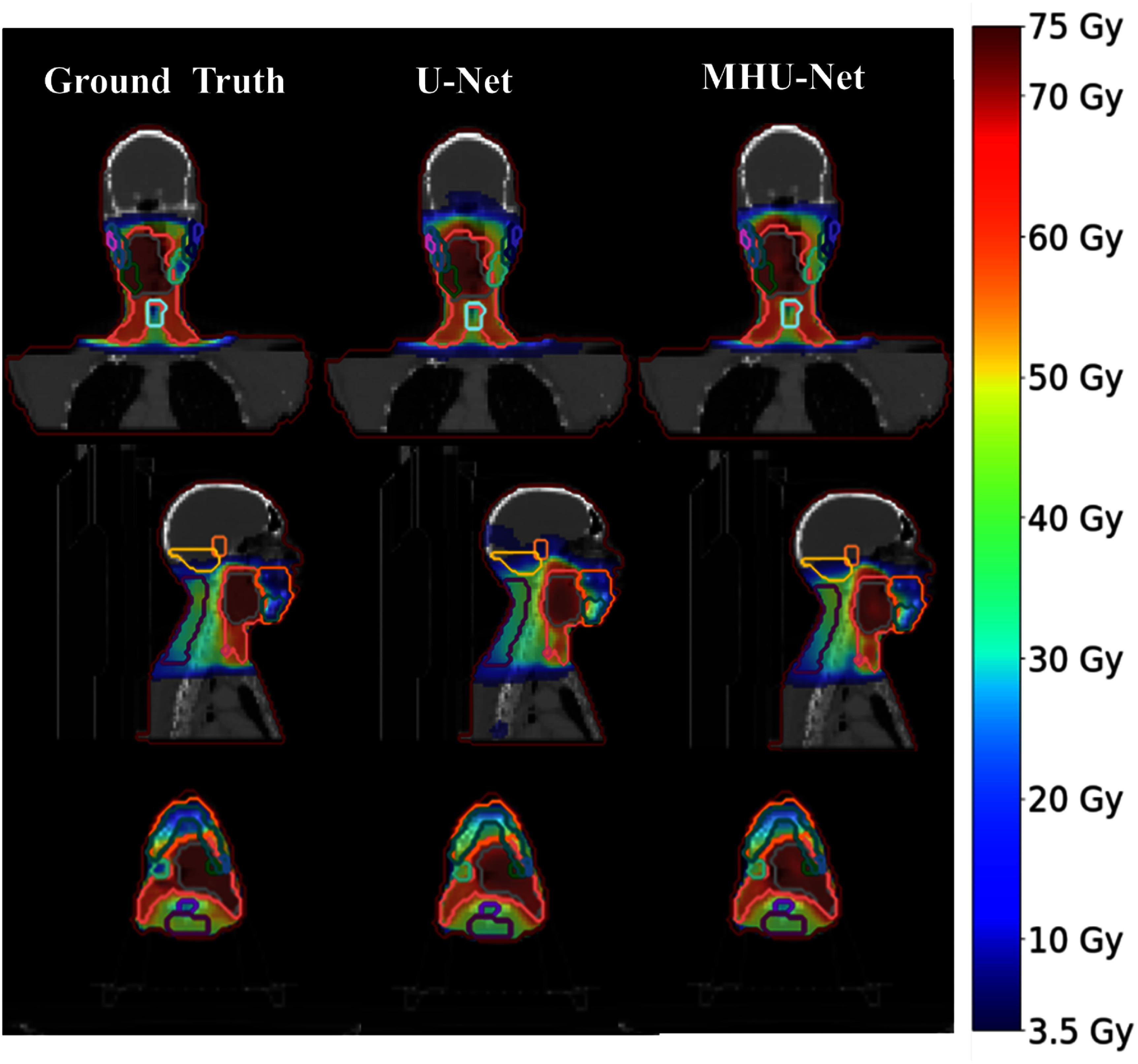
An example dose wash comparison between the ground truth and predicted dose distributions from U-Net and MHU-Net for a randomly selected adaptive session from the test dataset. The left column displays the ground truth clinical dose, while subsequent columns show the dose predictions generated by U-Net and MHU-Net, respectively.

Beyond the aggregated results from 10 patients, we can also examine individual cases to illustrate model performance. For instance, analyzing a representative patient from this cohort provides deeper insights into how U-Net and MHU-Net predict distributions compared to the ground truth. Visually, figure 4 shows that predicted distributions from both MHU-Net and U-Net are similar to the ground truth, maintaining uniformity in trade-offs between the PTV and OARs. However, in low-dose regions, U-Net predicts slightly extra doses in areas, such as the right shoulder and brain, compared to both ground truth and MHU-Net.

**Figure 5.**
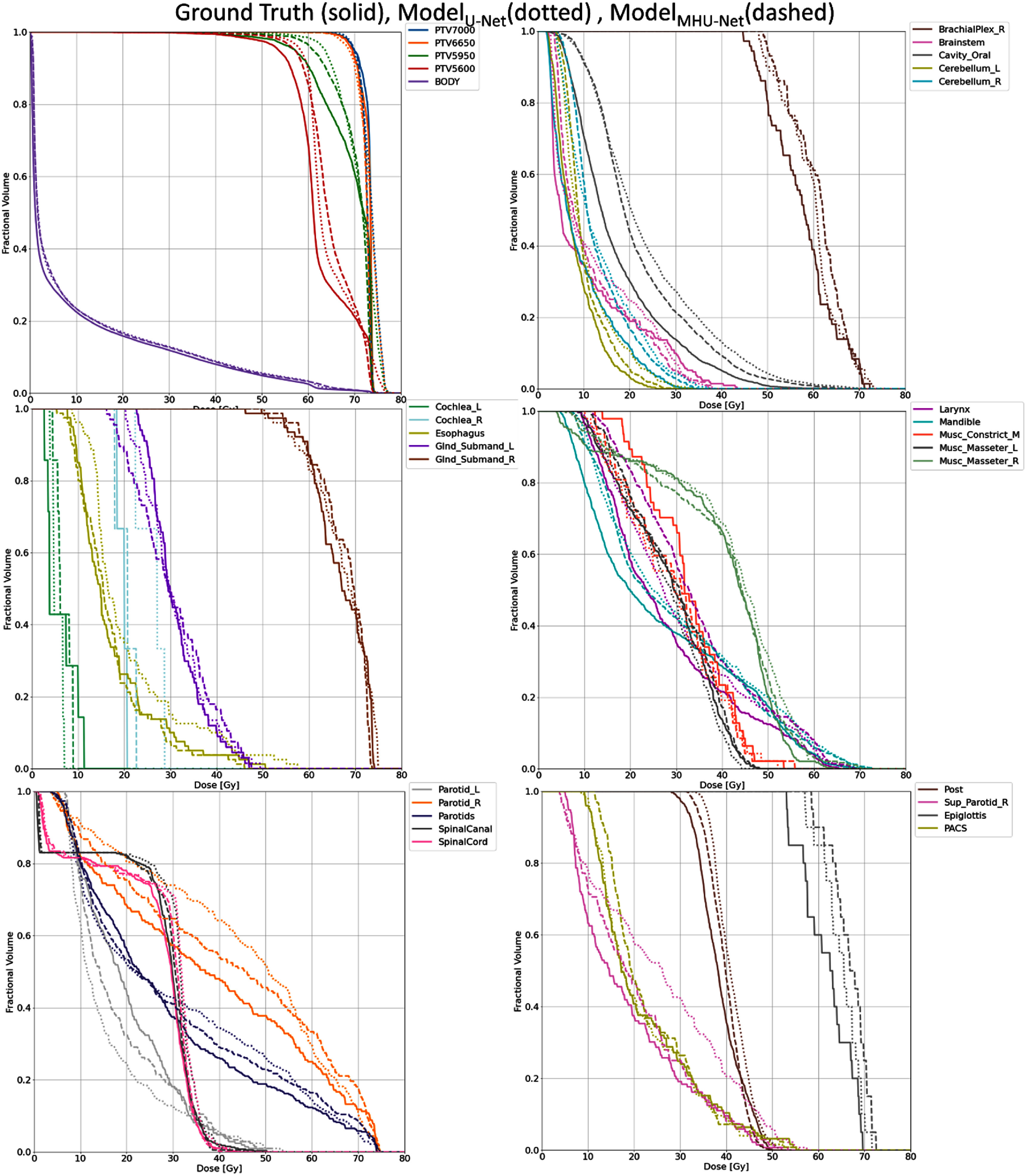
DVH comparisons for the PTV and OARs of the same test patient shown in figure 5. The solid lines correspond to the DVHs of the ground truth dose distributions, the dotted lines represent DVHs predicted by U-Net, and the dashed lines represent DVHs predicted by MHU-Net.

Figure 5 shows that MHU-Net predicts the PTV dose, specifically for PTV _5600_ and PTV _5950_, in the region corresponding to volume fractions of 0.95–0.99 (D95%–D99%) much more accurately, aligning closely with the ground truth on the DVH, whereas U-Net predictions show noticeable deviations. This suggests that MHU-Net more effectively ensures adequate target volume coverage, which is crucial for successful treatment. Furthermore, in the tail region of the PTV DVH, specifically around volume fractions < 0.02 (D2%), where U-Net consistently overestimated dose by 2%–3%, suggesting that MHU-Net also improves prediction accuracy in maximum dose regions, reducing hotspot overestimation For OAR dose predictions on the DVH in figure 5, MHU-et shows a closer agreement with the ground truth for the brainstem, oral cavity, left and right cerebellum, left and right cochlea, esophagus, mandible, left and right masseter muscles, left and right parotid glands, spinal canal, spinal cord, superior parotid, and posterior neck region. This observation is consistent with figure 5, where these structures exhibit shorter red error bars in the D_mean_ and D_max_ percentage error plots for MHU-et predictions across all test patients.

Visual inspection reveals that overall, MHU-Net appears to more accurately reflect patient-specific considerations for sparing OARs, aligning with the physicians’ original treatment plan since the predicted DVH curves by MHU-Net show better agreement with the ground truth distributions’ DVH curves than the corresponding DVH curves by U-Net.

## 4. Discussion

Driven by the need to support ART with more efficient and generalizable dose prediction models, this study introduces an innovative DL-based MHU-Net model designed to predict three-dimensional dose distributions for head and neck cancer patients. The model enhances workflow efficiency by providing planners with consistent dose predictions that serve as a strong starting point for adaptive plan generation, supporting more streamlined and informed decision-making in radiotherapy. To the best of our knowledge, this approach has not been previously applied to DL-based dose prediction in ART. During adaptive sessions, the pre-treatment plan serves as a crucial reference point, allowing for the evaluation of anatomical changes in the patient and necessary adjustments to the treatment plan with patient-specific goals. Training the model with pre-treatment plans ensures that the adaptive plans remain consistent with the original treatment objectives set by the physician and maintain the accuracy of their initial treatment plan despite anatomical changes across each adaptive session.

In this study, we evaluated predicted dose distributions using both qualitative and quantitative analyses. We assessed the dosimetric performance of each model by computing key dosimetric metrics and analyzing deviations between predicted and actual dose distributions. On average, our proposed MHU-Net model reduced dose prediction errors for OARs by 0.97% in *D*_mean_ and 1.78% in *D*_max_, compared to U-Net, reflecting greater consistency with the ground truth plans. Numerous studies have reported that the parotid and submandibular gland volumes frequently decrease during treatment, while the spinal cord and brainstem maintain a stable volume but may shift in position relative to the external contour due to weight loss [5, 27–29]. This highlights the importance of accounting for anatomical variations to ensure predicted dose distributions remain consistent with pre-treatment objectives, especially in regions where dose to critical structures may vary. As illustrated in figures 3 and 5, MHU-Net effectively reduces prediction errors in these regions, indicating its potential to support more accurate and individualized dose predictions consistent with pre-treatment objectives. Beyond improvements in OAR dose prediction, MHU-Net demonstrates more accurate PTV dose estimation, with predictions more closely matching the ground truth. It also achieved better dose coverage and homogeneity than U-Net, as summarized in table 1. Figures 4 and 5 further illustrate these improvements, with dose wash images and DVH curves from a representative patient showing MHU-Net more accurately predicts the intended dose patterns compared to U-Net. By enhancing the population model with a MHU-Net structure, our method represents an advancement in ART, offering precision in treatment planning.

Our results are slightly higher, they remain within the general range reported by other DL–based methods for head and neck patients. For example, Nguyen *et al* [15] reported Dmax and Dmean errors of 6.3% and 5.1%, respectively, using a HD U-Net, while Grönberg *et al* [16] achieved 2.7% and 2.0% errors with a 3D Dense Dilated U-Net. Liu *et al* [17] also reported similar performance using a Cascaded 3D U-Net. However, it is important to note that direct comparisons are limited due to differences in patient cohorts and evaluated structures. Prior studies focused on non-adaptive plans with fewer OARs, while our study involved adaptive cases and a broader set of OARs, increasing prediction complexity. More importantly, the primary objective of our study was to assess whether incorporating pre-treatment plan information via an additional input head could improve prediction accuracy and better align the predicted dose distributions with clinically approved plans. To isolate this effect, we conducted a controlled comparison between MHU-Net and the standard U-Net. Given that models, such as HD U-Net, Dense U-Net, and Cascaded U-Net, all share the same U-Net backbone, our ‘add-pre-plan’ strategy can be readily integrated into these architectures as well in the feature.

Beyond architectural innovations, patient-specific approaches, such as the method proposed by Rigaud *et al* [18], adopt a fundamentally different modeling strategy tailored to the needs of ART. These models are designed to maximize individualized accuracy and adaptability by training a separate network from scratch using each patient’s initial treatment data. While this approach enables highly personalized dose predictions aligned with unique patient anatomies and clinical constraints, it comes at the cost of substantial computational resources and time, often requiring over an hour of training per patient. Such demands pose significant challenges for scalability and integration into routine clinical workflows. In contrast, our proposed MHU-Net provides a balanced alternative by preserving the generalizability of population-based models while incorporating physician-defined clinical objectives from pre-treatment plans. This design allows MHU-Net to align closely with clinical intent and adapt to patient-specific anatomical variations, without necessitating individualized retraining. As a result, it highlights that the primary advantage of our architecture does not necessarily lie in outperforming patient-specific models of Rigaud *et al* [18], but rather in its ability to enhance efficiency and generalizability.

However, it is important to address several limitations of our study. Firstly, the dataset in this study is relatively small, including only 43 eligible patients. This limited data size is due to the novelty of the Varian Ethos treatment machine and ART software in stark contrast to typical DL datasets that often contain hundreds of thousands of data points. A larger data size could potentially enhance the prediction model’s performance. Secondly, our model’s input data is restricted to the planning PTV, OARs, and CT images, distance maps, and dose distributions. This limited input scope may constrain the model’s prediction performance. To improve the personalization and overall performance of dose prediction models, it would be beneficial to incorporate additional information, such as beam energies and beam orientations. These additional inputs could improve dose prediction accuracy, especially because these beam settings rarely change throughout a patient’s course of ART. While expanding the input features is a consideration, there are also limitations in the current input structure. Specifically, current dose prediction models rely on specific OARs as input, so the input to MHU-Net comprises a total of 181 channels, which is relatively high, which can limit the model’s practicality and generalizability. In clinical practice, some required OARs may be deemed unnecessary or omitted based on patient-specific factors. In future work, we aim to develop strategies that minimize the number of input channels without compromising data quality, while also incorporating dose constraints prescribed by physicians into the prediction model.

## 5. Conclusion

This study demonstrates the potential of MHU-Net to enhance dose prediction accuracy for online ART by integrating both pre-treatment and adaptive plan data compared to U-Net. By aligning dose predictions with clinical treatment objectives and adapting to changes in patient anatomy, MHU-Net offers a promising approach to improving the personalization and effectiveness of ART, ultimately leading to better patient outcomes in H&N cancer treatment.

## Data Availability

The data cannot be made publicly available upon publication because they contain sensitive personal information. The data that support the findings of this study are available upon reasonable request from the authors.
